# Revealing the viral culprits: the hidden role of the oral virome in head and neck cancers

**DOI:** 10.1007/s00203-025-04270-x

**Published:** 2025-02-28

**Authors:** Abdelhakim Salem

**Affiliations:** 1 Department of Oral and Maxillofacial Diseases, Clinicum, Faculty of Medicine, University of Helsinki, Helsinki, 00014 Finland; 2 Translational Immunology Research Program (TRIMM), Research Program Unit (RPU), University of Helsinki, Helsinki, 00014 Finland; 3 Head and Neck Oncobiome Group, Clinicum, Faculty of Medicine, University of Helsinki, Helsinki, 00014 Finland

**Keywords:** Oral virome, Head and neck cancers, Oropharyngeal squamous cell carcinoma, Nasopharyngeal carcinoma, Human papillomavirus, Epstein–Barr virus, Human Herpesvirus

## Abstract

The oral viral microbiome (or virome), encompassing a diverse community of viruses within the oral cavity, has emerged as a significant yet underexplored factor in head and neck cancers (HNCs). This review synthesizes recent evidence linking the oral virome to head and neck carcinogenesis, particularly oropharyngeal and nasopharyngeal carcinomas—the most common virus-associated subtypes of HNCs. Beyond pathogenesis, the diagnostic and therapeutic implications of the oral virome are explored, including non-invasive salivary detection of viral biomarkers for early cancer diagnosis, the development of targeted antiviral therapies, and preventive vaccination strategies—exemplified by the success of HPV vaccines in reducing the incidence of oropharyngeal cancers. Despite these advancements, challenges persist, including technical limitations, the need for longitudinal studies, and the integration of multi-omics approaches. A comprehensive understanding of the oral virome could revolutionize cancer diagnostics, therapeutics, and prevention. Moving forward, collaborative interdisciplinary efforts will be essential to fully leverage virome research for improving HNC outcomes.

## Introduction

### Background on head and neck cancers

Head and neck cancers (HNCs), encompass a diverse group of malignancies arising in the oral cavity, oropharynx, nasopharynx, hypopharynx, and larynx. Collectively, these cancers among the most prevalent cancers worldwide, with an estimated 890,000 new cases and 450,000 deaths reported in 2022 alone (Bray et al. 2024). Among these, head and neck squamous cell carcinoma (HNSCC) represents the most common histological subtype, with the majority of cases originate in the oral (OSCC) and oropharyngeal (OPSCC) tissues. The global incidence and mortality rates of HNCs show significant regional variations, with particularly high prevalence reported in South-Central Asia, Southeast Asia, and parts of Europe (Bray et al. 2024; Chi and Day 2015; Chamoli et al. 2021).

This variation is largely attributable to differences in risk factor prevalence, including tobacco use, alcohol consumption, betel quid chewing, and high-risk human papillomavirus (HPV) infections, which predominantly contribute to the development of OPSCC (Warnakulasuriya 2009; Chi and Day 2015; Johnson et al. 2020). Nasopharyngeal carcinoma (NPC), another distinct subset of HNC, is strongly associated with Epstein-Barr Virus (EBV) infection, particularly in endemic regions such as Southern China and Southeast Asia (Young and Rickinson 2004). Recent evidence also implicates oral dysbiosis and chronic inflammation in the disease’s pathogenesis (Metsäniitty et al. 2021). However, a substantial proportion of cases occur in individuals without these well-established risk factors, suggesting the involvement of additional unidentified oncogenic drivers (Llewellyn et al. 2001).

Despite advancements in therapeutic strategies, the 5-year survival rates for HNCs remain relatively low, with notable regional variation. Among the most studied virus-associated HNC subtypes, OPSCC and NPC demonstrated variable survival patterns between European countries. A comprehensive prognostic study by EUROCARE-5 reported 5‐year survival rates of 38.7% for OPSCC and 49.0% for NPC across Europe (Gatta et al. 2015), emphasizing the persistent challenges in managing these malignancies. According to a recent NORDCAN-based study, survival outcomes also differ significantly among Nordic countries (Zitricky et al. 2024). In OPSCC, the highest 5-year survival in men is observed in Sweden (68.9%), while Finland reports the lowest (64.7%). Among women, Finland has the best survival rate (72.4%), whereas Denmark has the lowest (61.0%). For NPC, the highest male 5-year survival is seen in Norway (73.8%), while Sweden reports the lowest (61.5%). For women, Finland leads with a survival rate of 77.4%, while Sweden reports 66.9% (Zitricky et al. 2024).

These differences in survival trends highlight the need for enhanced research to identify additional viral contributors, optimize diagnostic biomarkers, and develop novel virus-targeted therapies. The significant improvement in OPSCC survival reflects, in part, the success of HPV vaccination and improved therapeutic approaches, while the ongoing challenges in NPC management emphasize the importance of further exploring the EBV-host interactions in head and neck carcinogenesis.

### The oral microbiome and virome

The oral cavity harbors one of the most diverse microbiomes in the human body, comprising bacteria, fungi, viruses, archaea, and protozoa (Dewhirst et al. 2010). While the bacterial component (oral bacteriome) has been extensively studied, the viral microbiome (oral virome) remains comparatively underexplored. The oral virome includes both eukaryotic viruses that infect human cells and prokaryotic bacteriophages that infect bacterial cells (Wylie et al. 2012).

High-throughput sequencing has facilitated the exploration of the oral virome’s composition and its dynamic interactions within the microbiome. The oral cavity offers a diverse landscape with both hard and soft tissues supporting a wide variety of viruses, primarily bacteriophages and some eukaryote viruses (Abeles and Pride 2014). Identified viral families include Herpesviridae, Papillomaviridae, and Anelloviridae, which may exist in a latent or replicate states, influencing not only the host cells but also microbial ecosystems (Wylie et al. 2014).

While oral bacteriome consists of free-living bacterial cells that contribute to oral health and disease through metabolic activity and biofilm formation (Dewhirst et al. 2010), the virome adds another layer of complexity. Unlike bacteria, viruses lack metabolic machinery and rely on host cells for replication (Mesri et al. 2014). Eukaryotic viruses have the potential to alter host cell function, modulate immune responses, and induce malignant transformation (Mesri et al. 2014). Bacteriophages, on the other hand, can impact bacterial population dynamics through lysis or lysogenic conversion, reshaping the microbial community structure (Shkoporov and Hill 2019). Additionally, the virome is highly personalized and dynamic, evolving with age, health status, and environmental exposures (Abeles et al. 2014). These distinctions between the bacteriome and virome are crucial for understanding their interactions with the host immune system and tumor microenvironment, with potential implications for oral health and the pathogenesis of HNCs.

### Rationale for reviewing the oral virome in head and neck cancers

The International Agency for Research on Cancer (IARC) estimates that 20% of cancer cases worldwide are attributable to infectious agents, mostly viruses (Bouvard et al. 2009). While the role of HPV in OPSCC is well-documented, and EBV is a known driver of NPC, the broader contributions of the oral virome in HNCs remains poorly studied. Research has traditionally focused on bacterial dysbiosis in HNSCC (Metsäniitty et al. 2024), leaving critical gaps in our understanding of how viral communities influence oncogenic processes (Radaic and Kapila 2021).

Emerging evidence suggests that oral viruses may contribute to carcinogenesis through mechanisms such as insertional mutagenesis, modulation of cell cycle regulation, and induction of chronic inflammation. Additionally, synergistic interactions between viruses and other microbial constituents may further promote a pro-tumorigenic environment, shaping disease progression (Mesri et al. 2014). Advances in metagenomic sequencing and bioinformatics now offer unprecedented opportunities to unravel the oral virome’s composition and its role in cancer biology.

This review aims to synthesize current knowledge on the oral virome’s role in HNCs. Given the limited tissue-specific virome data, particularly for oral squamous cell carcinoma (OSCC), this review incorporates insights from other HNSCC subtypes, such as OPSCC and NPC, to provide a broader perspective on viral contributions to carcinogenesis. This review examines the oral virome’s composition, methodologies for virome research, viral oncogenic mechanisms, potential diagnostics and therapeutic applications, and current limitations in virome studies. Understanding the contributions of the oral virome to HNCs could unlock new avenues for early detection, targeted therapies, and preventive strategies in virus-associated malignancies.

## Composition of the oral virome

### Types of viruses Present in the oral cavity

#### DNA viruses

Several DNA viruses are implicated in HNCs, with HPVs and herpesviruses being the most extensively studied.

HPVs are small, non-enveloped, double-stranded DNA viruses belonging to the Papillomaviridae family. Over 200 HPV genotypes have been identified, classified into high-risk and low-risk types based on their oncogenic potential (Bernard et al. 2010). High-risk HPVs, particularly HPV-16 and HPV-18, are well-established etiological agents in cervical cancer and have been increasingly associated with OPSCC (Mehanna et al. 2013). The prevalence of oral HPV infection varies but remains substantially lower than genital infection rates (Gillison et al. 2012). Persistent infection with high-risk types in the basal epithelial cells can result in the expression of viral oncoproteins E6 and E7, which inactivate central tumor suppressors p53 and Rb, thereby promoting cellular proliferation and potential malignant transformation (Yeo-Teh et al. 2018).

Herpesviruses, large, enveloped, double-stranded DNA viruses, are known for their ability to establish lifelong latency. Key members of the Herpesviridae family relevant to the oral diseases and HNCs include herpes simplex viruses (HSV-1 and HSV-2), EBV, and human herpesvirus 8 (HHV-8) (Grinde 2013). HSV-1 and HSV-2 are commonly associated with orofacial lesions and have been investigated for their potential role in HNSCC due to their capacity to induce genomic instability (Parker et al. 2006; von Stebut et al. 2024). Linked to malignancies such as NPC and Burkitt’s lymphoma (Prabhu and Wilson 2016), EBV DNA has been detected in OSCC, suggesting a possible role in oral carcinogenesis (Jalouli et al. 2012; Zebardast et al. 2021). HHV-8 (Kaposi’s sarcoma-associated herpesvirus), recognized as a class I human carcinogen by IARC, has been detected in laryngeal squamous cell carcinoma (Mohamadian et al. 2014). Further, it has been detected in the saliva of patients with past or current Kaposi’s sarcoma (Vieira et al. 1997). However, its role in other subtypes such as OSCC remains unclear, requiring further investigations.

In addition to these well-characterized viruses, metagenomic studies have identified emerging DNA viruses such as Torque Teno Virus (TTV)—a single-stranded, circular DNA virus. First isolated from a hepatitis patient, TTV prevalence has been reported to be significantly higher in salivary and tumor samples from HNSCC patients compared to healthy controls (Hettmann et al. 2016). However, its potential oncogenic role in head and neck carcinogenesis remain largely unknown, representing an area ripe for exploration.

#### RNA viruses

RNA viruses in the oral virome are less studied than DNA viruses, and their oncogenic potential remains an emerging field of inquiry. Enteroviruses are small, non-enveloped, single-stranded RNA viruses belonging to the Picornaviridae family. They include Coxsackieviruses, Echoviruses, and Enterovirus 71 (EV71), which are common pathogens in humans, mainly via fecal–oral route of transmission (Stalkup and Chilukuri 2002). While enteroviruses in the oral mucosa are primarily known for causing Herpangina (aphthous pharyngitis) and hand-foot-and-mouth disease, research on their direct involvement in oral cancer is still unexplored (Dommisch and Schmidt-Westhausen 2024). Interestingly, given their ability to infect and lyse cancer cells, enteroviruses have recently emerged as a promising oncolytic therapeutic strategy (Sakamoto et al. 2023; Ruan et al. 2023). Further studies are needed to elucidate their role in the oral virome and potential impacts on oral health.

### Methods for studying the oral virome

Studying oral viruses typically requires an integrated approach involving cultivation, purification, enumeration, and characterization techniques. These methods offer critical insights into viral biology, from propagation in host systems to precise identification and quantification. Table 1 outlines key techniques along with their features and limitations in virome research (Payne 2017; Sridhar et al. 2020).

**Table 1:** Summary of selected methods commonly used in virome research, outlining their key features and limitations

Methods	Description	Limitations/Considerations
**Propagating viruses**		
Generating Cell Cultures	Primary or immortalized cell cultures provide a controlled environment for virus replication. Cells are grown in nutrient-rich media under sterile conditions.	Some viruses require specific cell types for replication; contamination risks need careful handling.
Fertile Chicken and Duck Eggs	Used for propagating many viruses, especially for vaccine production (e.g., influenza). Viruses are inoculated into specific embryonic tissues or fluids.	Limited to certain viruses; genetic and phenotypic considerations in using animal-derived systems.
Organoid cultures	Three-dimensional “mini-organs” derived from stem or progenitor cells. They provide more physiologically relevant model and useful for studying viruses that require tissue-specific environments e.g., Norovirus and Influenza virus.	Higher cost than standard 2D cultures; technically demanding; difficulty to infect with viruses; model variability; lack of immune cell components.
**Purifying viruses**		
Differential Centrifugation	Sequential centrifugation steps separate viral particles from debris based on size and sedimentation rate.	May not achieve complete purification; overlapping particle sizes can reduce separation efficiency.
Density Gradient Centrifugation	A gradient of Sucrose or Cesium Chloride separates viruses by buoyant density, resulting in highly purified viral particles	Time-consuming; requires specialized equipment and gradients may disrupt fragile viral particles.
**Counting viruses**		
Plaque Assays	Quantifies infectious virions by counting clear zones (plaques) formed in a monolayer of susceptible host cells.	Requires cytolytic viruses; slow process; dependent on host cell susceptibility.
Focus-Forming Assays	Similar to plaque assays but detects infected cell foci using antibodies, suitable for non-cytolytic viruses.	Antibody specificity is critical; requires appropriate detection reagents and longer assay times.
Endpoint Dilution Assays	Determines the virus dose (e.g., Tissue Culture Infectious Dose 50, or Lethal Dose 50) required to infect or kill 50% of test cells, animals, or systems.	Results are endpoint estimates, not absolute quantifications; time-consuming and resource-intensive.
Chemical/Physical Methods	Includes direct visualization of virions via electron microscopy, hemagglutination to detect viral binding to red blood cells, serological assays, and genome quantification via Polymerase Chain Reaction techniques	Hemagglutination may fail with non-hemagglutinating viruses; EM requires high expertise and expensive instruments.
**Visualizing viruses**		
Optical Microscopy	Limited resolution for viruses, but can detect cytopathic effects such as inclusion bodies or changes in cell morphology.	Cannot directly resolve viral particles; relies on indirect observations of infected cells.
Fluorescence Microscopy	Uses fluorescent tags to visualize viral proteins or particles, enhancing contrast and enabling specific localization studies.	Requires labeled reagents and specific probes; high background fluorescence can complicate interpretation.
Electron Microscopy	High-resolution imaging to visualize viral morphology using negative staining or thin-sectioning techniques.	Requires advanced training and expensive instruments; sample preparation can distort structures.
Cryo-Electron Microscopy	Preserves native viral structures by freezing samples rapidly, allowing high-resolution imaging and 3D reconstruction.	Extremely expensive; requires highly skilled operators and precise sample preparation to avoid artifacts.
**Characterizing viruses**		
Hemagglutination Assay	Detects viruses that agglutinate red blood cells, forming a visible lattice structure in the assay plate.	Only works with viruses that bind red blood cells; does not quantify virus infectivity.
Hemagglutination Inhibition Assay	Identifies antibodies that inhibit viral binding to red blood cells, useful for serotyping and vaccine efficacy studies.	Requires specific antibodies and careful controls; sensitivity depends on reagent quality.
Enzyme-Linked Immunosorbent Assays	Detects viral proteins or antibodies in samples using enzyme-linked antibodies and a colorimetric substrate.	Requires well-validated antibodies and controls; limited to detecting known viruses or antigens.
Western Blots	Confirms viral protein identity by separating proteins via gel electrophoresis and detecting them with specific antibodies.	Time-consuming; requires high-quality antibodies; low sensitivity for poorly expressed proteins.
Immunohistochemistry	Localizes viral antigens in tissue sections using enzyme-linked antibodies that produce colored precipitates for visualization.	May miss low-abundance viruses; cross-reactivity of antibodies can affect staining specificity.
Polymerase Chain Reaction	Amplifies specific regions of viral nucleic acids for detection, identification, or quantification.	Sensitive to contamination; requires specific primers and knowledge of viral genome sequences.
High Throughput Sequencing	Provides unbiased sequencing of viral genomes, enabling comprehensive analysis of known and novel viral species.	Expensive and data-intensive; requires robust bioinformatics tools for proper analysis and interpretation.

## Mechanisms of viral-induced carcinogenesis

**Fig. 1:**
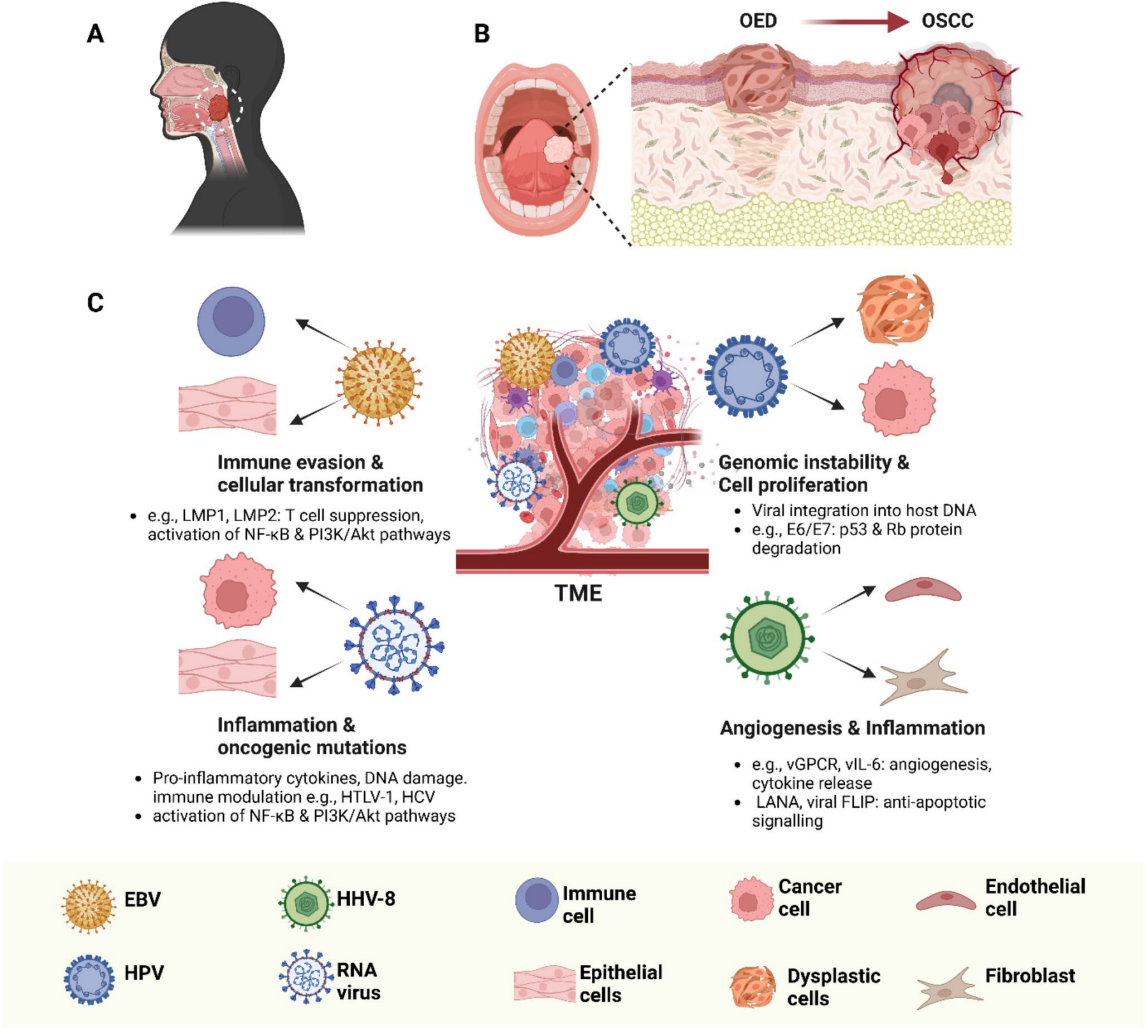
Simplified schematic overview of selected viral-mediated oncogenic mechanisms in head and neck cancers. (**A**) Most virus‐associated head and neck malignancies arise in the oropharynx and nasopharynx, where oncoviruses such as Epstein–Barr virus (EBV) and human papillomavirus (HPV) are frequently implicated. (**B**) Although oral squamous cell carcinoma (OSCC) is less commonly associated with viral infections, it typically develops via a multi‐step process from oral epithelial dysplasia (OED) to established carcinoma, reflecting the interplay of genetic and environmental risk factors. Ongoing research may further elucidate any potential viral contributions in OSCC (**C**) Selected mechanisms by which viruses contribute to tumor initiation and progression by altering the tumor microenvironment (TME), including immune evasion, cellular transformation, genomic instability, and aberrant proliferation. Key viral strategies involve modulation of host immune responses (e.g., LMP1/LMP2 of EBV), disruption of tumor suppressor pathways (e.g., HPV E6/E7‐mediated p53/Rb degradation), and induction of pro‐inflammatory signals that drive oncogenic mutations and angiogenesis. Color‐coded cell types (immune, endothelial, fibroblast, epithelial/dysplastic, and cancer cells) highlight the complex TME in which viral infection can influence malignancy development and progression

Viruses play a multifaced role in head and neck carcinogenesis, contributing to tumor initiation, progression, and immune evasion through diverse molecular mechanisms. While significant research has focused on mechanisms involved in HPV- and NPC-driven HNSCC, data specific to other common subtypes like OSCC remain limited. Nonetheless, viral-induced oncogenesis commonly involve direct interference with host genomic stability, promotion of chronic inflammation, and modulation of the tumor microenvironment (Fig. 1).

### Oncogenic viruses and their pathways

#### Human papillomavirus (HPV)

High-risk HPV types, especially HPV-16 and HPV-18, are well-documented drivers of OPSCC (Chaturvedi et al. 2011). The oncogenic potential of HPV is primarily mediated through the viral E6 and E7 oncoproteins, which promote genomic instability and uncontrolled proliferation. E6 promotes the degradation of the tumor suppressor protein p53 via the ubiquitin-proteasome pathway, which impairs DNA repair mechanisms and apoptosis, leading to genetic instability (Scheffner et al. 1990). The other viral oncoprotein E7 binds to the retinoblastoma protein, disrupting the association between cellular differentiation and proliferation in normal epithelium and allowing for viral replication. The integration of HPV genome into the host cell is a critical event that stabilizes the expression of E6 and E7, promoting malignant transformation (McLaughlin-Drubin and Münger 2009; Münger et al. 1989).

#### Epstein-barr virus (EBV)

EBV—a gamma-herpesvirus and reportedly the first identified human tumor virus—is associated with various malignancies, including NPC and lymphomas. In the oral cavity, EBV DNA and its associated proteins have been detected in oral hairy leukoplakia, oral potentially malignant disorders, and different anatomical subtypes of HNSCC including OSCC (Küppers 2003; Walling et al. 2003; Jalouli et al. 2012; Kikuchi et al. 2016; Strzelczyk et al. 2022), suggesting a possible role in oral tumorigenesis. EBV exhibits essential oncogenic proteins including Latent Membrane Protein 1 (LMP1). This 356-amino acid integral membrane protein acts as a constitutively active receptor, activating signaling pathways such as NF-κB, PI3K/Akt, and JAK/STAT, leading to cell proliferation and survival (Kaye et al. 1993). Epstein-Barr Nuclear Antigen 1 (EBNA1) is essential for viral genome maintenance and has been shown to induce genomic instability and inhibit apoptosis (Gruhne et al. 2009). Further, EBV can modulate the host immune response and induce immune escape, thus altering the microenvironment to favor carcinogenesis (Wang et al. 2022).

#### Human herpesvirus 8 (HHV-8)

HHV-8 is the etiological agent of Kaposi’s sarcoma and certain lymphoproliferative disorders (Ganem 2010). Although HHV-8 role in HNSCC is poorly defined, it warrants attention due to its oncogenic properties (Mohamadian et al. 2014; Vieira et al. 1997). Latent transcripts such as latency-associated nuclear antigen (LANA), viral cyclin, viral FLIP and viral-encoded microRNAs, enhance cell proliferation and prevent apoptosis, whereas viral interleukin-6 and viral chemokines promote pro-cancerous angiogenesis and inflammation (Mesri 2010).

### Viral integration and genetic instability

Integration of viral genomes into host DNA is a key driver of malignancy, disrupting cellular regulatory pathways and promoting carcinogenesis. Importantly, the integration of high-risk HPV DNA disrupts viral regulatory genes and leads to overexpression of E6 and E7 oncoproteins, which exploit the cellular ubiquitin–proteasome system to degrade retinoblastoma protein, driving the host cell into the S phase of the cell cycle, allowing viral replication and activating oncogenes (Yeo-Teh et al. 2018). Studies indicated that HPV integration occurred in 43–60.7% of HPV-positive HNSCC cases, encompassing both OSCC and OPSCC (Vojtechova et al. 2016; Koneva et al. 2018). Viral integration frequently occurs at fragile sites in the human genome—regions prone to breaks and rearrangements—such as FRA4B, FRA1F, FRA5G, FRA17B and FRA19A (Vojtechova et al. 2016). Furthermore, integration can lead to deletions, amplifications, and translocations, contributing to genomic instability. However, it remains uncertain whether HPV integration occurs prior to E6/E7-induced genetic instability or rather is a consequence of the instability itself (Häfner et al. 2008; Satgunaseelan et al. 2022; Koneva et al. 2018).

Unlike HPV, EBV generally maintains its genome as an episome, though its interaction with the host can indirectly induce DNA breaks and genetic instability, particularly in NPC (Fang et al. 2009). EBNA1, for instance, has been shown to impair DNA and inhibit repair mechanisms in the infected cells (Gruhne et al. 2009). Retroviruses, though not extensively studied in HNSCC, can also integrate into the host genome as part of their life cycle. Such integrations are often benign, however, some can lead to insertional mutagenesis and activation of oncogenic pathways (Warming et al. 2003).

### Immune evasion and chronic inflammation

Viruses have evolved sophisticated mechanisms to evade host immunity, facilitating persistent infections and chronic inflammation—both of which can promote carcinogenesis. HPV oncoproteins E6 and E7 downregulate the expression of interferons and other immune mediators (Park et al. 2000), while E5 protein has been shown to reduce antigen presentation by downregulating MHC class I molecules (Ashrafi et al. 2005). Similarly, EBV persists in host cells by balancing viral replication and immune evasion, for instance, by interfering with antigen processing and presentation through LMP1 and LMP2. The immune evasion capacity of EBV has been comprehensively reviewed by Zhao et al. (2024).

Persistent viral infections can lead to chronic inflammatory states marked by the continuous production of cytokines (e.g., IL-4, IL-6, VEGF, SDF-1) and reactive oxygen species. This pro-inflammatory microenvironment fosters DNA damage, uncontrolled cell proliferation, and angiogenesis, ultimately driving tumor progression (Grivennikov et al. 2010).

## Potential diagnostic and therapeutic implications

**Fig. 2:**
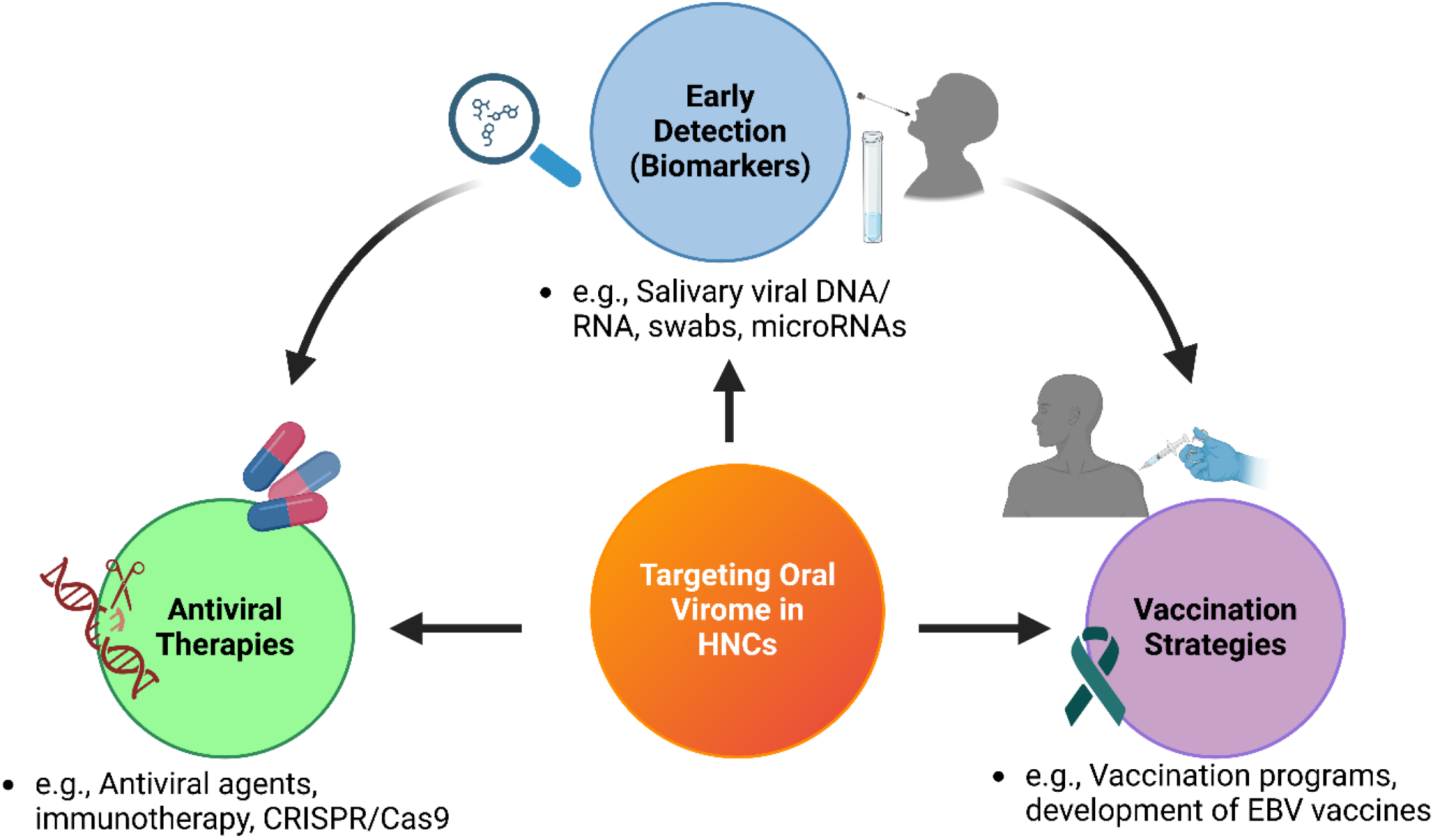
Conceptual overview of strategies to target the oral virome in head and neck cancers (HNCs). This figure outlines a multifaceted approach to mitigating virus-associated head and neck carcinogenesis. Early detection involves non-invasive salivary diagnostics, such as assays for viral nucleic acids and microRNAs, enabling rapid identification of at-risk individuals. Antiviral therapies encompass conventional antiviral agents, immunotherapies, and advanced gene-editing technologies such as CRISPR/Cas9, which are currently under investigations, aimed at suppressing or eliminating oncogenic viruses and their associated pathways. Vaccination strategies focus on preventing virus-driven malignancies, exemplified by prophylactic HPV vaccines and ongoing efforts to develop EBV-targeted vaccines. Arrows illustrate the interconnectivity and dynamic progression from detection to prevention and therapeutic intervention, highlighting a comprehensive framework for managing virus-associated HNCs

The complex interplay between the oral virome and carcinogenesis has opened new avenues for developing diagnostic, therapeutic and preventive strategies. Importantly, targeting viral components could offer opportunities to improve early detection, treatment and prevention of HNCs (Fig. 2).

### Viral biomarkers for early detection

The identification of viral biomarkers presents a promising avenue for the early detection of HNCs. Salivary diagnostics have gained attention in studies on virus-associated HNSCC, due to their non-invasive nature of sample collection and potential for real-time disease monitoring. In this regard, growing number of investigations have revealed that oncogenic-HPV-DNA can be detected in saliva samples obtained from HPV-positive OPSCC patients, offering opportunities for improved cancer diagnosis, treatment monitoring, and prognostic evaluation (Tang et al. 2019; Qureishi et al. 2018). In addition to viral DNA, HPV-derived microRNAs (miRNAs) have emerged as potential biomarkers (Wang et al. 2015). For instance, Wald et al. (2011) reported that miR-363, miR-33, and miR-497 were upregulated in HPV-positive HNSCC cells, while eight miRNAs (miR-155, miR-181a, miR-181b, miR-29a, miR-218, miR-222, miR-221, and miR-142-5p) were downregulated compared with HPV-negative cancer cells and normal oral keratinocytes. Furthermore, the HPV-16 E6 oncogene was found to alter miRNA expression, reinforcing its role in HNSCC progression (Wald et al. 2011). Collectively, miRNAs could help elucidate the role of HPV in HNSCC and be new biomarkers for the early detection of HNSC.

Similarly, EBV-encoded miRNAs hold diagnostic and prognostic potential. Lu et al. (2020) reported that circulating levels of miR-BART7-3p and miR-BART13-3p exhibited excellent diagnostic performance in NPC patients. Additionally, combining plasma levels of miR-BART7-3p with EBV DNA at diagnosis and post-radiotherapy provided valuable prognostic information, aiding in stratification of patients based on their risk of distant metastasis (Lu et al. 2020). These findings highlight the diagnostic and prognostic potential of viral biomarkers, however, further validation in large-scale, multi-center clinical studies is necessary before integrating these biomarkers into routine clinical practice.

### Antiviral therapies

Antiviral therapies in HNCs have gained increasing attention due to the established etiological role of oncogenic viruses, particularly HPV and EBV, in these malignancies. Consequently, antiviral strategies that disrupt viral oncogenesis or augment anti-viral immune responses have the potential to improve clinical outcomes in cancer patients.

One strategy involves therapeutic gene silencing to inhibit HPV oncogenes, thereby restoring normal cell cycle control and promoting apoptosis (Knoff et al. 2014). Preclinical studies using RNA interference (RNAi)-based approaches to silence E6/E7 have demonstrated reduced tumor suppression in HPV-positive HNSCC model, effectively reducing tumor proliferation and enhancing apoptosis in vivo (Kampel et al. 2021). Additionally, CRISPR/Cas9 gene editing system targeting HPV16-E7 DNA showed success in inducing apoptosis and inhibiting tumor growth in HPV-positive cell lines, with minimal effects on HPV-negative cells (Hu et al. 2014). Although still in early stages, gene-editing tools hold promise as future therapies for patients harboring high-risk HPV.

Small-molecule antiviral agents have also been explored. Cidofovir (CDV), abroad-spectrum antiviral drug, has been investigated for reducing viral load and inhibiting several HPV-induced benign and malignant hyperproliferations (Murono et al. 2001; Mertens et al. 2016). Interestingly, CDV significantly inhibited the growth of HPV-positive and -negative HNSCC cell lines, with more profound effect in HPV-positive tumors (Verhees et al. 2019). Clinical trials are ongoing to evaluate the effectiveness of antiviral drugs in conjunction with conventional therapies. Immunotherapeutic approaches are also being investigated. Therapeutic vaccines designed to elicit immune responses against viral oncoproteins, such as HPV E6 and E7, have demonstrated promising results in preclinical studies (Wang et al. 2024). These vaccines aim to boost the host’s immune system to recognize and eliminate virus-infected cells.

For EBV-associated HNCs, antiviral drugs like ganciclovir have been used with limited success due to the EBV’s latent state in tumor cells (Soldan et al. 2022). Therefore, strategies aimed at reactivating latent viruses—rendering them susceptible to antiviral therapies—are under investigation. These developments signify the potential of antiviral-based strategies in HNC treatment, though further clinical validation is required for their widespread implementation.

### Vaccination strategies

Preventive vaccination remains one of the most effective strategies against oncogenic viruses. The introduction of prophylactic HPV vaccines has significantly reduced the incidence of cervical cancers and shows promise for preventing HPV-related cancers, including OPSCC (Herrero et al. 2013; Chaturvedi et al. 2018). Studies have demonstrated that the quadrivalent and nonavalent HPV vaccines elicit robust immune responses against high-risk HPV types associated with head and neck carcinogenesis (Bergman et al. 2019).

To maximize public health benefits, vaccination coverage must be expanded, particularly among adolescents and young adults. In June 2020, the United States Food and Drug Administration (FDA) expanded the indications for the 9-valent HPV vaccine for the prevention of OPSCC and other HNCs. Thus, based on vaccine efficacy data from cervical cancer, a gender-neutral vaccination programs could maximize the preventive impact on HPV-associated OPSCC and reduce its incidence among young and middle-aged adults by 2045 (Zhang et al. 2021; Diana and Corica 2021).

Research is also ongoing to develop vaccines against EBV. While no licensed EBV vaccine is currently available, candidate vaccines targeting EBV glycoproteins have entered clinical trials (Li et al. 2024). A successful EBV vaccination could potentially reduce the incidence of EBV-associated malignancies, presenting an important preventive measures in HNC control.

## Challenges and future directions

### Technical limitations in virome research

Despite significant advancements, several technical limitations hinder the full understanding of the oral virome’s role in carcinogenesis. One of the major challenges is the detection of low-abundance viruses within the complex oral microbiome. Given their limited volume, clinical samples such as oral swabs and saliva rinses often contain low viral loads with a high background of host and bacterial DNA, making virome characterization difficult (Khan Mirzaei et al. 2021; Rascovan et al. 2016). In addition, commonly employed sampling and processing methods can introduce biases. For instance, the use of small filter pores (e.g., 0.2 μm membranes) may inadvertently overlook larger viruses, leading to underrepresentation of certain viral populations. Similarly, homogenization with larger ceramic beads (e.g., 2.8 mm) can disrupt viral particles, irrespective of homogenization speed (Conceição-Neto et al. 2015). Unlike bacteria, viruses lack a universal marker, such as the 16 S ribosomal RNA used in the bacterial microbiome studies, making virome identification in mixed samples particularly challenging. Standard sequencing techniques often fail to detect rare viral species or struggle to assemble complete viral genomes (Roux et al. 2017), limiting our ability to fully characterize the virome’s diversity.

Another key limitation is the differentiation between latent and active viral infections. Many oncogenic viruses, including EBV and HPV, establish latency, where viral gene expression and replication are minimal or highly restricted for prolonged periods. Consequently, determining their accurate contribution to oncogenesis remains challenging using conventional methodologies (Young and Rickinson 2004; Woodman et al. 2007). Improving detection sensitivity and specificity requires the development of enhanced bioinformatics tools and enrichment techniques. A further complicating matter is the need to differentiate causation from association. Only part of people infected with any of the human tumor viruses develop tumors; the mere presence of viral DNA does not necessarily indicate a direct role in carcinogenesis, necessitating functional studies to establish causality (Münger and Howley 2002; Moore and Chang 2010).

### Need for longitudinal studies and multi-omics approaches

Longitudinal studies are crucial for understanding the temporal relationship between viral infections and cancer development. Most current microbiome studies are cross-sectional, providing only snapshots of viral prevalence. Long-term cohort studies would enable researchers to observe the progression from initial viral infection to malignant transformation. Such studies could also identify potential windows for intervention and improve the understanding of viral persistence, immune responses, and co-factors involved in carcinogenesis (Franco et al. 1999; Rodríguez et al. 2010). Establishing biobanks and standardized protocols for sample collection and analysis would facilitate these efforts.

Integrating multi-omics approaches offers a comprehensive view of the interactions between the host, viruses, and other microbiota (Zhang et al. 2010). Combining genomics, transcriptomics, proteomics, and metabolomics can reveal complex networks and pathways involved in head and neck carcinogenesis (Rai et al. 2018). Proteomic profiling can identify viral proteins and host responses, providing insights into mechanisms of immune evasion and oncogenesis (Slebos et al. 2013). Moreover, integrating microbiome data can elucidate synergistic effects between viruses and other microorganisms. Such holistic approaches may identify novel biomarkers and therapeutic targets, ultimately advancing personalized oncology.

## Conclusions

The oral virome plays a significant yet underexplored role in HNCs. Oncogenic viruses such as HPV and EBV facilitate disease initiation and progression through intricate mechanisms such as immune evasion, genomic instability, and pro-inflammatory pathways. Recent advances in salivary virome diagnostics, along with emerging targeted antiviral and vaccine-based strategies, underscore the tangible clinical potential of harnessing virome data. Nonetheless, major gaps persist, including technical hurdles in virus detection and the need for longitudinal, multi‐omic investigations. Integrating virome analysis into standard HNC research paradigms is imperative to develop refined diagnostic tools, targeted therapies, and robust preventive measures. As the field progresses, collaborative efforts spanning epidemiology, molecular biology, and clinical research will be essential to fully exploit virome insights for improved cancer patient outcomes.

## Data Availability

No datasets were generated or analysed during the current study.
